# Males benefit more from cold water immersion during repeated handgrip contractions than females despite similar oxygen kinetics

**DOI:** 10.1186/s12576-020-00742-5

**Published:** 2020-03-05

**Authors:** Jiří Baláš, Jan Kodejška, Dominika Krupková, David Giles

**Affiliations:** 1 Faculty of Physical Education and Sport, Charles University Prague, José Martího 31, 16252 Prague 6, Czech Republic; 2 Lattice Training Ltd., Chesterfield, Derbyshire UK

**Keywords:** NIRS, Forearm, Recovery, Haemoglobin, Tissue oxygenation

## Abstract

The purpose of the present study was to assess the effect of different water immersion temperatures on handgrip performance and haemodynamic changes in the forearm flexors of males and females. Twenty-nine rock-climbers performed three repeated intermittent handgrip contractions to failure with 20 min recovery on three separate laboratory visits. For each visit, a randomly assigned recovery strategy was applied: cold water immersion (CWI) at 8 °C (CW8), 15 °C (CW15) or passive recovery (PAS). While handgrip performance significantly decreased in the subsequent trials for the PAS (*p* < 0.05), there was a significant increase in time to failure for the second and third trial for CW15 and in the second trial for CW8; males having greater performance improvement (44%) after CW15 than females (26%). The results indicate that CW15 was a more tolerable and effective recovery strategy than CW8 and the same CWI protocol may lead to different recovery in males and females.

## Background

Repeated contractions of the forearms are required for many daily physical activities and forearm strength and endurance are known to be performance-limiting factors in sports, such as rock climbing and canoeing [11, 18]. It has been demonstrated that lower ambient temperature (10 °C) prolongs forearm flexor endurance during intermittent contractions [19] and that the optimal muscle temperature for sustained isometric contractions varies between 25 and 30 °C [3, 5]. However, while the association between muscle temperature and isometric contraction has been described extensively [3, 5, 26], the use of muscle cooling as an effective recovery strategy between repeated isometric contractions is not well documented. As many physical and sporting activities are often repeatedly practised for prolonged periods within the same day, muscle cooling has the potential to improve performance in such scenarios.

In sports practice, cold water immersion (CWI) following activity is often promoted in order to maintain repeat performance in hot environments, reduce muscle soreness, and/or aid recovery from secondary muscle damage [16, 25]. In a recent review, it was proposed that the dominant mechanism by which CWI facilitates short-term recovery is (1) via ameliorating hyperthermia, which is associated with central nervous system-mediated fatigue; (2) by reducing cardiovascular strain through the redistribution of blood flow from the periphery to the core which increases cardiac output; and (3) by faster removal of muscle metabolites [13]. However, this is only known to be valid for whole body endurance exercise in hot and humid environments. During and after resistance exercise, the main physiological mechanisms responsible for the benefits of CWI are not yet fully understood, and have recently been under debate [20, 21, 30]. Hyperthermia rarely occurs during resistance exercise, such as isolated isometric forearm contractions, rather fatigue is thought to mainly be associated with peripheral factors [3].

It is known that post-resistance exercise cooling decreases heart rate, induces greater parasympathetic activity, reduces microvascular perfusion, and reduces muscle swelling [17, 20, 30]. However, the effect of CWI techniques on subsequent resistance performance is not clear. While a number of studies assessing CWI and repeated performance consistently report a faster rate of muscle deoxygenation and decreased venous blood oxygen saturation after contraction, inconsistencies in the total amount of muscle oxygenation during the contractions were found [20, 21, 30]. The discrepancy between studies may be due to differences in the water temperature used, time between CWI and subsequent performance, or the velocity of contraction [20]. Therefore, before trainers and coaches can safely and effectively incorporate CWI recovery strategies, the effect of temperature with respect to the type of muscle contraction and the physiological response should be explored. Furthermore, the investigation of the effect of CWI on subsequent muscular performance using intermittent isometric contractions of the finger flexors, which induce local blood occlusion, appears more suitable for this purpose as the muscles work in relative isolation from the rest of the body [5].

Preliminary results from a study assessing the effect of two CWI recovery strategies (8 °C and 15 °C) on intermittent isometric forearm contractions, demonstrated the potential performance advantages of CWI over passive recovery [15]. Cold water immersion at 15 °C was found more beneficial than 8 °C. However, high individual variability in the results was reported and no explanation of the physiological mechanisms was provided. Sex differences may have contributed to large variability due to the differences in forearm strength, anthropometric characteristics and body composition in male and female athletes [2]. Anthropometric differences such as forearm muscle volume or body fat affect the gradient of muscle cooling, and therefore cold perception, microvascular response, and other physiological mechanisms during and after recovery [6]. Therefore, we hypothesised that the same cooling protocols may induce different haemodynamic response and subsequent performance changes in males and females. Consequently, the main aim of the present study was to assess the effect of different water immersion temperatures on handgrip performance and haemodynamic changes in the forearm flexors of males and females.

## Methods

### Experimental approach to the problem

**Fig. 1:**
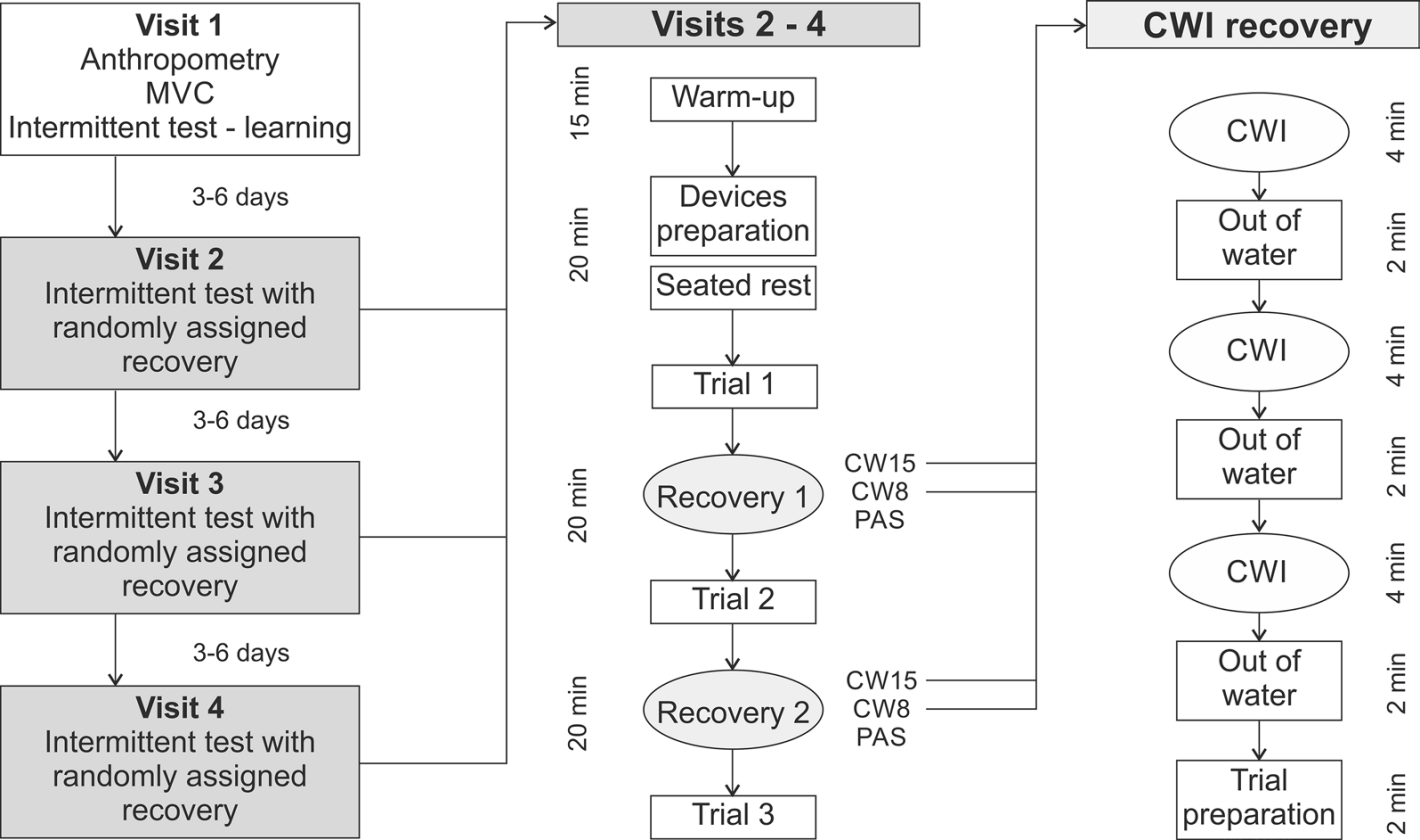
Design of the study. *MVC* maximal voluntary contraction, *CWI* cold water immersion, *CW15* cold water immersion at 15 °C water, *CW8* cold water immersion at 8 °C water, *PAS* passive recovery strategy

A within-subject two-factor experimental design (trials x water temperature), with sex as grouping variable, was used to determine the effect of different recovery strategies on performance and haemodynamic characteristics of males and females. On their first visit, participants provided informed consent, completed anthropometric measurement, and physical activity and health questionnaires. After the warm-up, maximal finger strength was determined, the intermittent endurance test was described, and familiarisation trials conducted. During the next three visits, each separated by 3–6 days, participants completed one of the following three recovery interventions in a random order: (1) passive recovery (PAS); (2) cold water immersion at 8 °C (CW8); (3) cold water immersion at 15 °C (CW15) (Fig. 1).

Each visit began with the same 15-min warm-up, which consisted of cardiovascular (5 min walking stairs) and mobilisation exercises and short intermittent contractions of finger flexors with a progressive increase in intensity till 80% of maximum voluntary contraction (MVC) was achieved. After the configuration and placement of the near-infrared spectroscopy (NIRS) optodes and thermistor sensors 10 min of seated rest was provided, to assess participants resting haemodynamic response. Three intermittent handgrip trials until volitional fatigue were then completed, interspersed by 20 min of the randomly selected recovery intervention (Fig. 1). To ensure that participants attended the laboratory in a similar state, they were asked to refrain from any strenuous activities 24 h, caffeine for 12 h, and food 3 h prior to testing. All testing was conducted in the afternoon between 14.00 and 18.00.

### Participants

Thirty-four athletes who regularly took part in upper body and finger flexors strength training were recruited to perform repeated intermittent handgrip contractions to failure. As such, 17 males (age 27.7 ± 10.2 years;) and 17 females (age 26.3 ± 4.6 years) with rock climbing experience at an intermediate to advanced ability level volunteered [4]. Athletes were healthy non-smokers who were not taking any vascular acting medication. The study conformed to the recommendations of the local Ethics Committee in accordance with the Declaration of Helsinki. All participants were informed of the experimental risks and provided informed consent prior to the commencement of data collection.

### Finger strength and endurance tests

To test for finger flexor strength and endurance, a custom-made dynamometer (3D-SAC, Spacelab, Sofia, Bulgaria) was used [18]. The dynamometer enabled the participants to control the intensity and duration of each contraction. Further, the device provided real-time feedback using both visual and acoustic signals. The dynamometer was calibrated for a hold 23 mm deep which maximised the activation of the flexor digitorum profundus (FDP) [23].

During the first visit, the MVC of the dominant finger flexors was assessed twice (separated by two minutes). On presentation of an acoustic signal, participants were asked to progressively pull on the hold as hard as possible for 5 s. Participants were verbally encouraged to achieve maximal effort throughout. The highest value from the two trials was used to determine their relative workloads (60% MVC) in the proceeding endurance trials. MVC and endurance tests were determined whilst standing with the shoulder flexed to 180° and the elbow fully extended to represent an ecologically valid test [18] and to prevent blood pooling [27].

The intermittent handgrip endurance test was performed at 60% MVC, with a work:relief ratio of 8:2 s. The test started with an acoustic signal and participants were provided with visual feedback to ensure the correct force was applied. If the force dropped by more than 10% for more than 1 s, the test was automatically halted. An acoustic signal, as well as the visual display, marked the start and end of each contraction/relief period. Participants were required to achieve the desired target force as quickly as possible. Only the time in the target zone was used in the analysis. Moreover, due to sex differences in forearm strength, the force–time integral (FTI) was calculated as the mean force in the target zone multiplied by the time of the test. To assess the velocity of contraction, the rate of force development was calculated from the time to achieve 50% of the target force (N.s^−1^).

### Recovery strategy

Participants were instructed to sit in the same position with knees and hips at 90° flexion. The tested arm placed at 20° shoulder abduction and 15° flexion, the elbow at 80° flexion and the wrist slightly pronated. During CWI the forearm, from elbow to mid-palm was immersed in water, while during PAS participants remained seated in their chair in the same anatomical position. Each CWI recovery protocol consisted of three 6-min cycles (4 min immersion, 2 min out of water) and 2 min for preparation (Fig. 1). This intermittent CWI protocol was based on the previous forearm recovery study with positive effect on intermittent handgrip performance [12]. Water temperature was regulated by ice cubes throughout the whole procedure, to within ± 1 °C and agitated to avoid water heating at the skin’s surface. At the end of the last CWI, participants were asked to assess subjective perception of the procedure (agreeable, neutral, and disagreeable) and water temperature on 7-point scale (cold − 3; neutral 0; warm 3).

### Skin temperature

Water, skin and ambient temperature were measured by thermistor sensors and recorded on a data logger S0141 (Comet system, Rožnov pod Radhoštěm, Czech Republic). The thermal sensor for skin temperature was attached distal to the NIRS optodes and sealed from water ingress using adhesive tape. The intra-muscular temperature was not recorded during this study.

### Near-infrared spectroscopy

NIRS optodes were placed in 1/3 of proximal distance between medial epicondyle of humerus and carpus. The FDP was located by a chartered physiotherapist using the technique suggested by Schweizer and Hudek [23], where the thumb and the first finger were squeezed together. The flexor was then palpated to locate the middle of the muscle belly. Optodes were placed in a secure holder on the skin using bi-adhesive tape. The holder was fixed using dark tape to prevent contamination from ambient light. To ensure no variability in optode placement between sessions, pen marks were used on the skin.

Continuous wave NIRS (Oxymon, Artinis Medical System, BV, Netherlands) was used to assess the oxygenation and blood volume changes. Two emitting optodes were spaced 4 cm and 3.6 cm from one receiver, respectively. This configuration enables the calculation of absolute ratios of oxyhaemoglobin (O_2_Hb), deoxyhaemoglobin (HHb) using the spatially resolved spectroscopy approach. Tissue saturation index (TSI) was determined as O_2_Hb/(HHb + O_2_Hb). Absolute concentrations of total haemoglobin (tHb) were estimated from the path-length factor, which was fixed to 4.0, as suggested by van Beckvelt et al. (2002) for forearm measurements. The sampling frequency was set to 10 Hz and data were sent via optical cables to a personal computer, where online recording and all parameters were undertaken using the Oxysoft software (Artinis Medical System, BV, The Netherlands). Forearm skinfold thickness (under the NIRS optode) was measured using a skinfold calliper (Harpenden, Baty, West Sussex, UK). Adipose fat was not considered to affect the NIRS signal as skinfold measures were below the limits set by Van Beekvelt et al. [27] (1/2 of the skinfold = 1.7 ± 0.5 and 2.0 ± 0.7 mm for males and females, respectively). Only TSI and tHb were included in the analysis as the measures of tissue oxygenation and blood volume [10]. Resting tHb and TSI were determined using the last minute of 10 min of seated rest. Changes in tissue oxygenation and blood volume (perfusion) during exercise (∆ TSI, ∆ tHb) were calculated from all 8-s contractions and 2-s relief periods, respectively. Moreover, the mean minimum concentrations of TSI (TSI_min_) from the contraction periods were used in the analysis as the indicator of tissue deoxygenation [9].

### Statistical analysis

Mean ± standard deviation (SD) in tables and 95% confidence intervals (95% CI) in figures were used to characterise performance and NIRS characteristics. Temperature and pain perception during recovery was assessed using median and frequency distribution for each recovery period. A mixed factorial ANOVA (3 × 3×2; test trial × recovery strategy × sex) was calculated to assess the effect of different recovery strategies and sex on forearm muscles performance and haemodynamic response during exercise. To evaluate haemodynamic response and skin temperature during recovery periods, mixed factorial ANOVAs were applied at the beginning, 4th, 6th, 10th, 12th, 16th, and 18th minute, corresponding to the time of forearm immersion and emersion. The mean values from the 20-s interval were taken in the analysis. If significant, Tukey’s tests were applied. Pearson correlation coefficients were calculated to assess the relationship between performance changes and possible covariates. Differences between sexes were analysed using t-test and Cohen delta (*d*). Statistical significance was set to *P* < 0.05. Effect size for ANOVA was calculated using partial eta squared (µ_p_^2^). Mean detectable change, as stated previously [1], was taken for a criterion of practical effect. All calculations were completed using IBM SPPS 24.0 (IBM, Armonk, New York).

## Results

Due to drop out and technical errors, five participant’s data were excluded. In total, 15 males and 14 females were included in the results. Males (M) were significantly taller, with greater body mass, less body fat, larger forearm circumference, greater handgrip strength, and shorter duration of Trial 1 than females (F) (Table 1).

**Table 1:** Anthropometric and performance characteristics in male and female athletes

	Males	Females	Differences		
	Mean ± SD	Mean ± SD	95% CI	Sig	*d*
Body mass (kg)	71.0 ± 9.3	56.9 ± 5.5	− 20.0 to − 8.2	*P < 0.001*	1.35
Body height (cm)	178.3 ± 9.7	166.1 ± 5.6	− 18.3 to − 6.1	*P* <* 0.001*	1.22
Body fat (%)	7.6 ± 2.0	16.4 ± 3.5	6.6 to 11.0	*P < 0.001*	3.08
Forearm skinfold (mm)	3.5 ± 0.5	4.0 ± 0.7	0.08 to 1.1	*P = 0.023*	0.72
Forearm circumference (cm)	28.1 ± 2.2	24.2 ± 1.3	− 5.3 to − 2.5	*P* <* 0.001*	2.16
Climbing ability (IRCRA scale)	18.0 ± 4.0	15.3 ± 3.2	− 5.4 to 0.2	*P* = 0.065	0.74
*F*_max_ (N)	571 ± 85	386 ± 71	− 25.0 to − 12.8	*P < 0.001*	2.36
*F*_max rel_ (Nkg^−1^)	8.1 ± 1.3	6.8 ± 1.3	− 0.24 to − 0.02	*P = 0.020*	1.00
Time of the trial 1 (s)—mean of the three visits	83.5 ± 23.5	105.7 ± 21.7	2.3 to 36.1	*P* =* 0.014*	0.98
FTI of the trial 1 (Ns)—mean of the three visits	26,431 ± 7118	21,946 ± 4848	− 990 to − 106	*P* = 0.059	0.73

Italic designates significant differences at *P* < 0.05

*SD* standard deviation, *CI* confidence interval, *d* Cohen’s delta, *FTI* force–time integral, *F*_*max*_ maximal finger flexor strength, *F*_*max rel*_ maximal finger flexor strength related to body mass, *IRCRA* International Research Rock Climbing Association

### Intermittent handgrip test

**Fig. 2:**
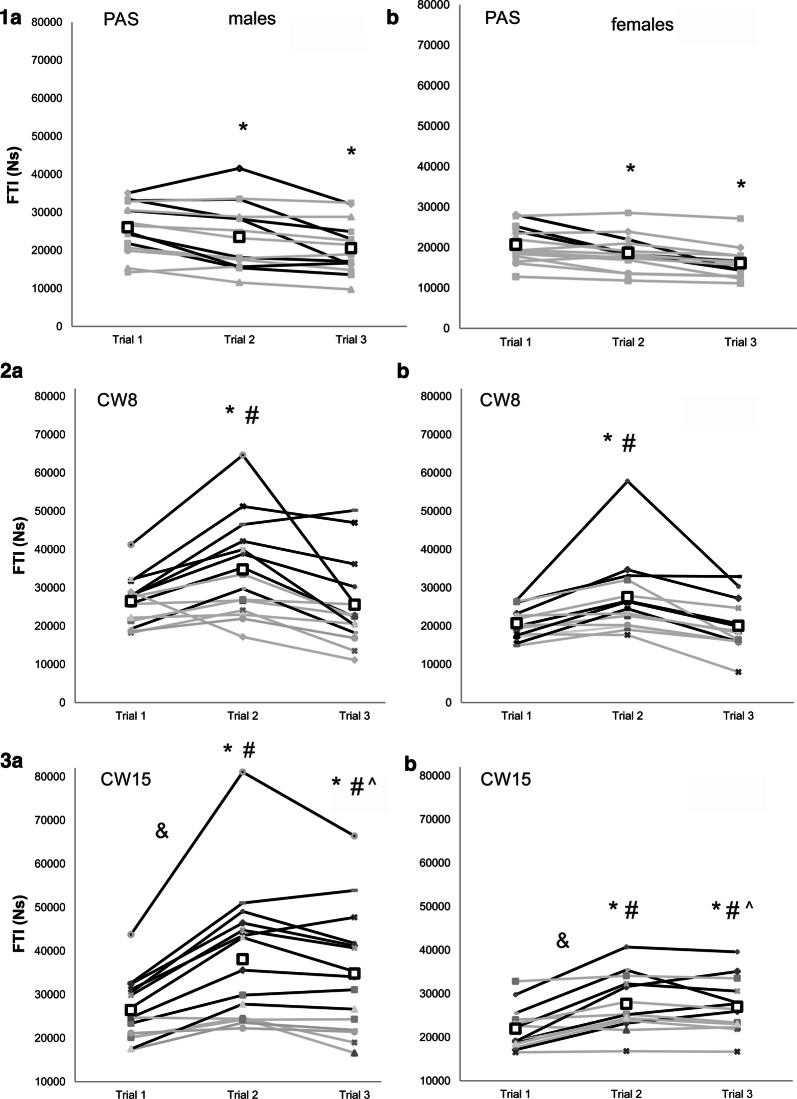
Handgrip performance (FTI force–time integral) during: (1) passive recovery (PAS); (2) cold water immersion at 8 °C (CW8); and (3) at 15 °C (CW15) in males (a) and females (b). Bold rectangles designate the mean of the trial. Black lines represent athletes with performance changes exceeding minimal detectable change (6323 Ns). *statistically different from the Trial 1 at *P* < 0.05; #statistically different from the PAS at *P* < 0.05; ^statistically different from the CW8 at *P* < 0.05; &significant interaction between males and females at *P* < 0.05

The FTI significantly decreased in the second (↓10% for both F and M) and third trial (F ↓22%; M ↓21%) for the PAS recovery strategy. For the CW8, the FTI increased in the second (F ↑33%; M ↑31%) but decreased in the third trial (F = M ↓3%). For the CW15, there was a significant FTI increase in the second and third trial (F ↑26%; M ↑44% and F ↑23; M ↑32 compared to baseline, respectively). A significant interaction (*P* = 0.007, *µ*_p_^2^ = 0.24) between PAS and CW15, trial 1 and 2 and males and females was found. After CW15, 10 males improved over the minimal detectable change (6323 Ns), however, this was only true for 6 females (Fig. 2). There was no significant change in the rate of force development after any of the recovery strategies (Additional file 1: Tables S1 and S2).

**Fig. 3:**
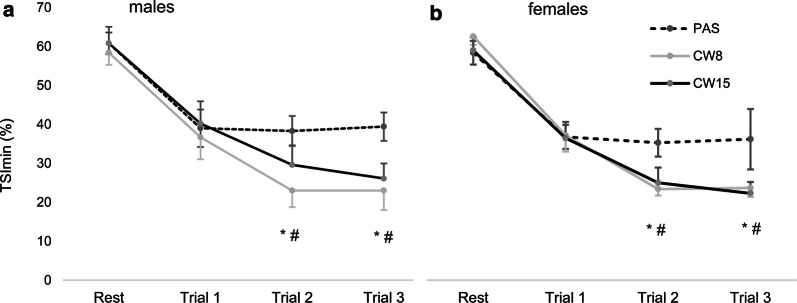
Mean (± *95% *confidence interval) decrease of tissue saturation index from resting values to minimal contraction values (TSI_min_) after passive recovery (PAS), cold water immersion at 8 °C (CW8) and 15 °C (CW15) in males (**a**) and females (**b**). *CW8 statistically different from PAS at *P* < 0.05; #CW15 statistically different from the PAS at *P* < 0.05

No interaction was found for mean tHb, ∆tHb or ∆TSI during contraction (8 s) and relief (2 s) phases (Additional file 1: Tables S1 and S2). However, minimum TSI during contractions decreased significantly more from rest values after CWI than after PAS for both males and females (Fig. 3).

The FTI improvement from trial 1 to trial 2 after the CW15 recovery strategy was related to performance, anthropometric and haemodynamic characteristics (Table 2). Only initial performance, climbing ability level and perceived cold temperature were significantly related to performance changes. Together, they explained 64% of variability in the performance change, based on a linear regression model (Additional file 1: Figure S1). Surprisingly, no haemodynamic parameter accounted for performance change.

**Table 2:** The relationship (Pearson correlation coefficient—R) between handgrip performance increase (∆FTI) from the trial 1 to trial 2 after 15 °C cold water immersion and performance, anthropometric and haemodynamic characteristics of participants

Variable	R	*P*
Climbing performance (IRCRA scale)	*0.528*	*0.003*
Finger strength max (N)	0.329	0.082
FTI trial 1 (Ns)	*0.704*	* < 0.001*
Time trial 1 (s)	0.278	0.144
*∆* TSI_contraction_ trial 2−deoxygenation (%)	− 0.086	0.658
*∆ *TSI_relief_ trial 2−re-oxygenation (%)	0.026	0.894
TSI_min_ trial 2 (%)	0.283	0.137
THb_mean_ trial 2 (%)	0.329	0.082
Perceived temperature (7 point scale)	*0.633*	* < 0.001*
Pain and cold scale (3 point scales)	0.043	0.823

Italic designates significant differences at *P* < 0.05

*FTI* force–time integral, *TSI *tissue saturation index, *TSI*_min_ lowest value of TSI during contraction,* tHb* total haemoglobin, *tHb*_mean_ average *tHb* value during contraction

### Recovery

**Fig. 4:**
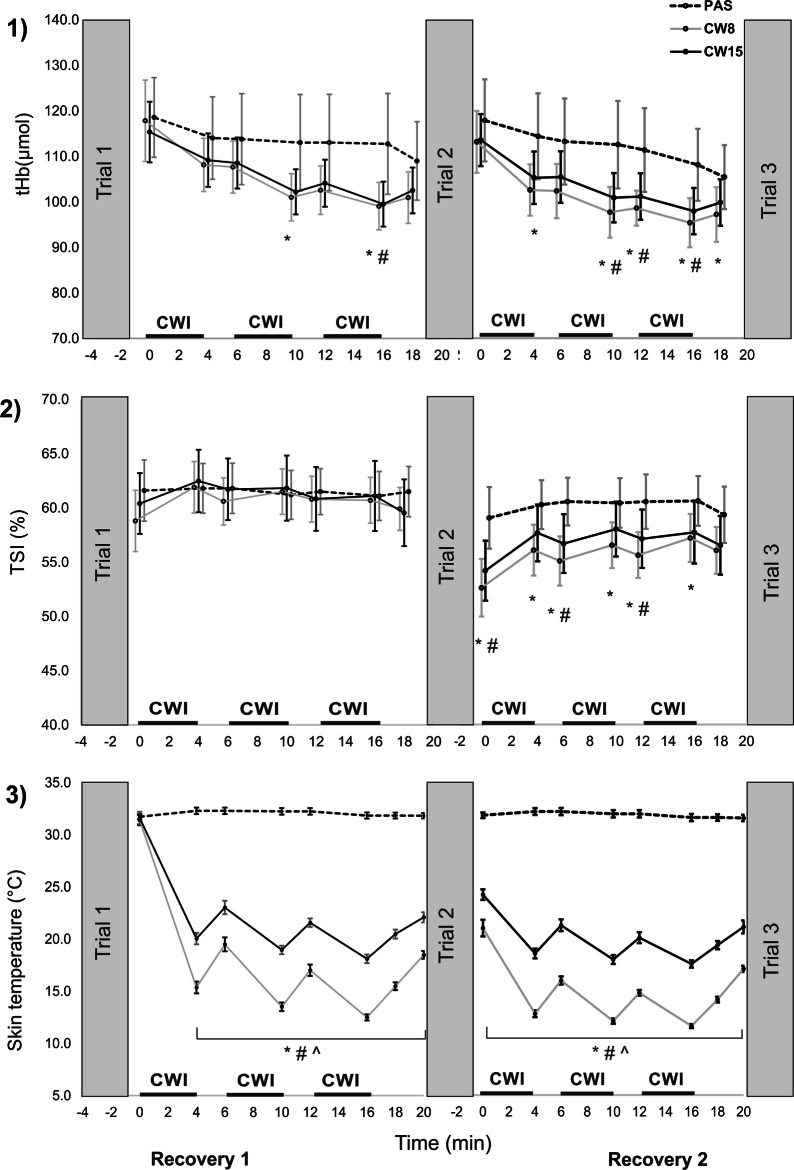
Mean (± 95% confidence interval) (1) total haemoglobin changes (tHb); (2) tissue saturation index (TSI) in flexor digitorum profundus; and (3) skin temperatures during passive recovery (PAS), cold water immersion at 8 °C (CW8) and at 15 °C (CW15) after repeated handgrip exercise to failure. Bold horizontal lines indicate cold water immersion (CWI) time. There were no differences between males and females, their results are summarised together.*CW8 statistically different from PAS at *P* < 0.05; #CW15 statistically different from the PAS at *P* < 0.05; ^CW8 statistically different from the CW15 at *P* < 0.05

During all recovery periods, similar results were found for males and females, therefore both are summarised together (Fig. 4). THb decreased from ~ 120 mmol to ~ 100 mmol, indicating lowered perfusion under the inspected area at the end of each recovery period, with respect to post-test values. Although tHb was lower for both CWI strategies compared to PAS recovery strategies (mean difference ranging approx. from 5 to 15 µmol), the difference was under the determined MDC value (39.6 µmol).

TSI did not differ statistically during the first recovery period, however, it was significantly lower for both CWI strategies than for the PAS recovery strategy during the second recovery period (Fig. 4). The mean difference ranged from ~ 3 to ~ 7% during the second recovery period which was under the MDC stated for TSI at rest (13.6%).

The skin temperature did not change after PAS, fluctuating around 32 °C. With the CW15 strategy there was a temperature drop to ~ 18–20 °C after immersion and temperature increased to ~ 21–24 °C after emerging the forearms from the water (Fig. 4). With CW8, the forearm skin temperature dropped to ~ 12–15 °C after immersion and increased to ~ 14–19 °C after emerging (Fig. 4).

### Pain and cold scale

CW8 was rated mostly as disagreeable, while CW15 was evaluated as neutral or agreeable (Table 3). The median of perceived temperature (cold − 3, 0 neutral; warm + 3) after CW8 was − 2 after both recovery periods, however, only 0 and − 1 with CW15 after the first and second recovery period, respectively (Table 3). Generally, males better tolerated the colder temperatures and perceived them as less cold than their female counterparts (Table 3).

**Table 3:** Perception of pain during CWI (agreeable, neutral, and disagreeable) and water temperature on 7 point scale (from cold − 3; neutral 0; to warm 3) in total sample *N* = 29 (F − females *N* = 14; M − males *N* = 15)

	Cold perception (F;M)	Pain perception (frequency distribution − number) (F;M)		
	(median from − 3 to 3)	Agreeable	Neutral	Disagreeable
CW8—1st recovery	− 2 (− 3; − 2)	0 (0;0)	5 (2;3)	24 (12;12)
CW8—2nd recovery	− 2 (− 2.5; − 1)	0 (0;0)	11 (3;8)	18 (11;7)
CW15—1st recovery	0 (0;0)	12 (5;7)	15 (2;8)	2 (2;0)
CW15—2nd recovery	1 (− 0.5; − 1)	11 (2;9)	13 (8;5)	5 (4;1)

## Discussion

The main aim of the present study was to assess differences in handgrip performance and haemodynamic changes during repeated intermittent handgrip contractions following different CWI recovery strategies. The uniqueness of this study is that isolated local repeated isometric contractions were undertaken under three different temperatures conditions in male and female climbers with control for haemodynamic response during exercise and recovery. The results demonstrate that CWI (1) attenuated the decrease in intermittent handgrip performance to failure in both males and females; (2) prolonged time to failure in the subsequent tests; and, (3) led to a lower muscle oxygen saturation during contractions. Moreover, CW15—inducing skin temperatures of ~ 18–20 °C—was more tolerable and effective recovery strategy than CW8—leading to a skin temperature of ~ 12–15 °C, while males benefited more from CW15 than females despite similar haemodynamic response.

The increase in the subsequent number of contractions after CWI was surprising considering that much of the previous CWI literature has found either similar exercise performance to baseline [12, 30] or an attenuated decrease when compared to other recovery strategies [21]. Possible physiological mechanisms are discussed in the text, particularly with respect to sex differences in forearm muscle performance.

A recent study proposed that the benefits of CWI are due to hydrostatic pressure, rather than water temperature, which decreases arterial and cutaneous blood flow; and that cool water (22 °C) may be an equally effective recovery strategy as cold water (8 °C) [17]. From the current data, we can conclude that hydrostatic pressure was not the main mechanism leading to performance improvements, as there were substantial performance differences in the third trial following both CWI recovery strategies. Moreover, perfusion during recovery was lower for both CWI strategies, indicating vasoconstriction of muscle and cutaneous circulation with respect to PAS. This vasoconstriction was accompanied by statistically lower TSI in the 2nd recovery period, demonstrating lower muscle oxygenation (balance between capillary perfusion and O_2_ supply and O_2_ extraction). The decreased TSI was not seen during the 1st recovery, possibly due to higher muscle temperature at the beginning of cooling period. Differences in the haemodynamic response between the 1st and 2nd recovery show that muscle temperature, rather than hydrostatic pressure, are responsible for TSI changes. However, the differences in tHb and TSI between PAS and CWI recovery strategies were small and did not exceed the minimal detectable changes stated for this type of measurement [1].

During exercise, absolute values and changes in tHb were similar for all three recovery strategies in males and females, indicating that muscle blood perfusion was not affected by the low temperature. It may be that microvascular response was quickly restored during exercise in response to the metabolic demands of the muscle. Similarly, the amount of tissue deoxygenation and re-oxygenation were the same after all recovery strategies. Interestingly, after both CWI recovery strategies the absolute values of TSI dropped to a greater degree (TSI_min_) than after PAS recovery strategy. This may be interpreted as greater capillary O_2_ extraction in the cooled muscle given that muscle perfusion was similar and may be one of the potential mechanisms leading to greater submaximal performance. For instance, greater TSI drops during sustained handgrip contractions were found in upper-body strength-trained athletes, indicating more efficient oxygen utilisation of the forearm muscles [9]. Similarly, CWI may induce changes associated with more effective oxidative pathways [29]. Nevertheless, greater TSI decrease cannot be the only mechanism leading to performance improvements as there was a performance decrease in the 3rd trial after CW8 despite low *TSI*_min_. Moreover, the decrease in *TSI*_min_ was not related to any performance increase (Table 2).

The subjective pain and temperature perception during recovery may have played a role in subsequent performance differences in males and females. The majority of participants assessed CW8 as disagreeable and very cold, while CW15 was rated as thermally neutral or slightly cold and agreeable or neutral. Females generally assessed the CWI as less tolerable than males, which has been probably related to their lower forearm circumference, and greater cooling gradient than in their males. It has been suggested that muscle temperatures between 25–30 °C are optimal, in order to maximise the number of repeated isometric contraction before reaching fatigue [3, 5, 24]. While too high muscle temperatures are associated with higher use of glycolysis and metabolites accumulation; muscle temperature under 25 °C may interfere with nervous or neuromuscular transmission in some fibres and lead to lowered nerve conduction velocity, changes in intra- and extracellular ion concentrations, and prolonged contraction–relaxation time [3, 5, 22]. Lowered muscle temperature may explain the drop of performance in the 3rd trial after CW8 which was perceived as very cold and disagreeable. On the other hand, temperatures perceived as thermally neutral or slightly cold corresponded with positive effects on performance.

A particularly interesting finding of this study was the association between initial FTI and performance changes, however, the initial time of contraction demonstrated no relationship. Due to male’s greater MVC, understandably, their 60% MVC contractions were completed at a greater absolute strength for significantly shorter time (by ~ 20%) in Trial 1 than their female counterparts. It is plausible that males exhaustive trials invoked type II fibre recruitment and fast glycolytic pathways to restore ATP to a greater extent than the female participants. Moreover, reported climbing performance, which relies on maximal strength and high-intensity forearm contraction, was also related positively with performance changes. Consequently, these findings suggest that cooling strategies may have more benefits on subsequent performance in exercise with greater Type II fibres recruitments. This interpretation is supported by several studies demonstrating positive effect of cooling on mechanical efficiency and glyco- and glygogenolysis sparing effect [5, 7, 31].

The authors acknowledge several limitations. There was no control for muscle temperature and the effect of body fat, forearm circumference, and/or thermoregulation on the cooling gradient is not known. Nevertheless, no relationship was found between anthropometric variables, including forearm skinfold thickness, and performance changes. Secondly, the menstrual cycle might have confounded the results for females. The phase of the cycle was recorded, analysed and no effect was confirmed. xzThirdly, only local isometric contractions in relatively standardised conditions were used. Although the isolated local contractions may be more suitable for understanding physiological response on cooling, the transfer to complex sport movements is not straightforward as many other mechanisms that affect sport performance are involved. Finally, haemodynamic changes have high day-to-day variability and the use of NIRS has also several limitations [8, 14]. Therefore, we interpreted the results according to minimal detectable changes, which should minimise the error due to the biological variability.

## Conclusions

CWI attenuated performance decreases after repeated intermittent isometric handgrip contractions to failure to a greater extent than passive rest in both males and females. Moreover, while both CWI prolonged time to failure in the subsequent trial, CW15 inducing skin temperatures of ~ 18–20 °C was more tolerable and effective recovery strategy than CW8 leading to skin temperatures of ~ 12–15 °C. Males benefited more from CW15 than females, although they had similar haemodynamic response during exercise and recovery periods. Trials performed after CWI were accompanied by larger drop of TSI when compared to passive recovery. Although lowered forearm muscles oxygen saturation might have contributed to prolong time to failure, the changes in TSI were not related to performance increases and other physiological mechanisms must have been involved in the performance improvements seen. It was shown that temperature perception and the level of initial performance mostly affect subsequent exercise after CWI.

### Supplementary information

**Additional file 1: Table S1.** Time to failure, rate of force development (RFD), and forearm oxygenation characteristics during intermittent handgrip contractions in passive recovery (PAS), cold water immersion at 8°C (CW8) and 15°C (CW15) in females. **Table S2.** Time to failure, rate of force development (RFD), and forearm oxygenation characteristics during intermittent handgrip contractions in passive recovery (PAS), cold water immersion at 8°C (CW8) and 15°C (CW15) in males. **Figure S1.** Summary of stepwise liner regression model with handgrip performance increase (∆FTI) from the trial 1 to trial 2 after 15°C cold water immersion as dependent variable and performance, anthropometric and hemodynamic characteristics as independent predictors.

## Data Availability

The datasets used and/or analysed during the current study are available from the corresponding author on reasonable request.

## Abbreviations

ANOVA: Analysis of variance
CWI: Cold water immersion
CW8: Immersion at 8 °C water
CW15: Immersion at 15 °C water
F: Female
FDP: Flexor digitorum profundus
FTI: Force–time integral
HHb: Deoxyhaemoglobin
MDC: Minimal detectable change
MVC: Maximal voluntary contraction
M: Male
NIRS: Near-infrared spectroscopy
O_2_: Oxygen
O_2_Hb: Oxyhaemoglobin
PAS: Passive recovery strategy
tHb: Total haemoglobin
TSI: Tissue saturation index
