# Supplementary material for: Males benefit more from cold water immersion during repeated handgrip contractions than females despite similar oxygen kinetics
Source: J Physiol Sci. 2020 Mar 5;70:13. doi: 10.1186/s12576-020-00742-5 (PMC7058574; doi:10.1186/s12576-020-00742-5)
Supplement: Supplementary file 1 — Additional file 1: Table S1. Time to failure, rate of force development (RFD), and forearm oxygenation characteristics during intermittent handgrip contractions in passive recovery (PAS), cold water immersion at 8°C (CW8) and 15°C (CW15) in females. Table S2. Time to failure, rate of force development (RFD), and forearm oxygenation characteristics during intermittent handgrip contractions in passive recovery (PAS), cold water immersion at 8°C (CW8) and 15°C (CW15) in males. Figure S1. Summary of stepwise liner regression model with handgrip performance increase (∆FTI) from the trial 1 to trial 2 after 15°C cold water immersion as dependent variable and performance, anthropometric and hemodynamic characteristics as independent predictors. [file 12576_2020_742_MOESM1_ESM.docx]

**Additional digital content**

**Table S1** Time to failure, rate of force development (RFD), and forearm oxygenation characteristics during intermittent handgrip contractions in passive recovery (PAS), cold water immersion at 8°C (CW8) and 15°C (CW15) in females

|  | **Trial 1** | **Trial 2** | **Trial 3** |
| --- | --- | --- | --- |
| ***PAS*** |  |  |  |
| Time to failure | **100.4 ±23.9** | **89.2 ± 16.9*** | **76.9 ± 13.6*** |
| *Contraction (8 s)* |  |  |  |
| RFD (N.s^-1^) | 484.8 ± 171.6 | 477.7 ± 154.9 | 473.8 ± 144.1 |
| ∆ TSI (%) | 8.1 ± 2.6 | 8.7 ± 3.2 | 9.4 ± 3.0 |
| ∆ tHb (μmol) | 14.6 ± 8.0 | 16.0 ± 9.8 | 15.7 ± 8.2 |
| tHb_mean_ (μmol) | 94.7 ± 17.1 | 95.3 ± 16.6 | 92.4 ± 23.6 |
| *Relief (2 s)* |  |  |  |
| ∆ TSI (%) | 8.2 ± 3.1 | 8.9 ± 3.5 | 9.7 ± 3.4 |
| ∆ tHb (μmol) | 15.9 ± 8.2 | 17.3 ± 10.0 | 16.9 ± 8.4 |
| ***CW8*** |  |  |  |
| Time to failure | **100.7 ± 24.6** | **133.1 ± 49.8*#** | **98.1 ± 35.3** |
| *Contraction (8 s)* |  |  |  |
| RFD (N.s^-1^) | 475.1 ± 178.0 | 464.2 ± 110.8 | 414.9 ± 115.2 |
| ∆ TSI (%) | 8.6 ± 2.5 | 8.8 ± 1.8 | 8.4 ± 1.6 |
| ∆ tHb (μmol) | 12.7 ± 6.4 | 11.3 ± 5.5 | 9.8 ± 4.7 |
| tHb_mean_ (μmol) | 90.5 ± 9.3 | 89.6 ± 9.5 | 86.9 ± 9.5 |
| *Relief (2 s)* |  |  |  |
| ∆ TSI (%) | 8.8 ± 2.8 | 8.3 ± 2.0 | 7.2 ± 1.9 |
| ∆ tHb (μmol) | 14.0 ± 6.4 | 12.0 ± 5.6 | 10.4 ± 4.5 |
| ***CW 15*** |  |  |  |
| Time to failure | **105.7 ± 21.7** | **133.9 ± 34.5*#** | **131.0 ± 35.3*#^** |
| *Contraction (8 s)* |  |  |  |
| RFD (N.s^-1^) | 445.2 ± 112.9 | 450.3 ± 150.9 | 427.4 ± 132.5 |
| ∆ TSI (%) | 8.5 ± 2.1 | 8.7 ± 2.3 | 8.2 ± 2.2 |
| ∆ tHb (μmol) | 12.7 ± 4.9 | 11.8 ± 4.6 | 10.2 ± 3.6 |
| tHb_mean_ (μmol) | 93.5 ± 11.9 | 94.6 ± 10.9 | 91.7 ± 8.8 |
| *Relief (2 s)* |  |  |  |
| ∆ TSI (%) | 8.7 ± 2.2 | 8.6 ± 2.3 | 7.9 ± 2.2 |
| ∆ tHb (μmol) | 14.0 ± 5.3 | 12.7 ± 5.0 | 10.7 ± 3.6 |

Notes: RFD rate of force development; TSI tissue saturation index; TSI _min_ lowest value of TSI during contraction; tHb total haemoglobin; tHb_mean_ average tHb value during contraction

* statistically different from the Test 1 at *P*<0.05

# statistically different from the PAS recovery strategy at *P*<0.05

^ statistically different from the CW8 recovery strategy at *P*<0.05

**Table S2** Time to failure, rate of force development (RFD), and forearm oxygenation characteristics during intermittent handgrip contractions in passive recovery (PAS), cold water immersion at 8°C (CW8) and 15°C (CW15) in males

|  | **Trial 1** | **Trial 2** | **Trial 3** |
| --- | --- | --- | --- |
| ***PAS*** |  |  |  |
| Time to failure | **81.2 ± 20.4** | **73.4 ± 26.0*** | **64.0 ± 19.3*** |
| *Contraction (8 s)* |  |  |  |
| RFD (N.s^-1^) | 564.6 ± 151.8 | 593.8 ± 181.6 | 550.0 ± 160.7 |
| ∆ TSI (%) | 8.4 ± 1.7 | 9.1 ± 2.1 | 9.3 ± 2.1 |
| ∆ tHb (μmol) | 20.4 ± 11.5 | 20.5 ± 12.0 | 18.2 ± 9.0 |
| tHb_mean_ (μmol) | 110.1 ± 15.8 | 108.4 ± 14.1 | 106.1 ± 12.5 |
| *Relief (2 s)* |  |  |  |
| ∆ TSI (%) | 8.4 ± 1.7 | 9.0 ± 2.1 | 9.2 ± 2.4 |
| ∆ tHb (μmol) | 21.9 ± 11.9 | 21.5 ± 11.4 | 19.9 ± 8.9 |
| ***CW8*** |  |  |  |
| Time to failure | **83.2 ± 18.7** | **108.9 ± 39.1*#** | **79.9 ± 32.9** |
| *Contraction (8 s)* |  |  |  |
| RFD (N.s^-1^) | 546.4 ± 176.3 | 533.6 ± 145.7 | 519.9 ± 170.8 |
| ∆ TSI (%) | 8.3 ± 2.5 | 9.5 ± 2.3 | 9.6 ± 2.6 |
| ∆ tHb (μmol) | 15.7 ± 7.0 | 12.8 ± 4.1 | 14.6 ± 11.0 |
| tHb_mean_ (μmol) | 103.4 ± 16.3 | 101.5 ± 16.7 | 99.5 ± 17.4 |
| *Relief (2 s)* |  |  |  |
| ∆ TSI (%) | 8.2 ± 2.8 | 9.0 ± 2.3 | 8.6 ± 3.1 |
| ∆ tHb (μmol) | 17.0 ± 7.3 | 14.0 ± 4.5 | 15.5 ± 11.1 |
| ***CW 15*** |  |  |  |
| Time to failure | **83.5 ± 23.5** | **118.9 ± 47.7*#** | **109.4 ± 44.4*#** |
| *Contraction (8 s)* |  |  |  |
| RFD (N.s^-1^) | 529.7 ± 154.1 | 520.4 ± 108.0 | 521.7 ± 131.8 |
| ∆ TSI (%) | 7.6 ± 2.3 | 9.0 ± 2.2 | 9.5 ± 2.3 |
| ∆ tHb (μmol) | 15.9 ± 5.8 | 12.9 ± 4.0 | 14.0 ± 5.4 |
| tHb_mean_ (μmol) | 104.4 ± 16.8 | 104.0 ± 16.6 | 102.7 ± 16.1 |
| *Relief (2 s)* |  |  |  |
| ∆ TSI (%) | 7.5 ± 2.2 | 8.6 ± 2.0 | 9.2 ± 2.5 |
| ∆ tHb (μmol) | 17.1 ± 5.8 | 13.7 ± 4.1 | 14.9 ± 5.4 |

Notes: RFD rate of force development; TSI tissue saturation index; TSI _min_ lowest value of TSI during contraction. tHb total haemoglobin; tHb_mean_ average tHb value during contraction

* statistically different from the Test 1 at *P*<0.05

# statistically different from the PAS recovery strategy at *P*<0.05

^ statistically different from the CW8 recovery strategy at *P*<0.05

**Fig S1** Summary of stepwise liner regression model with handgrip performance increase (∆FTI) from the trial 1 to trial 2 after 15°C cold water immersion as dependent variable and performance, anthropometric and hemodynamic characteristics as independent predictors.

| Model | R | R Square | Adjusted R Square | Std. Error of the Estimate |
| --- | --- | --- | --- | --- |
|  |  |  |  |  |
| 1 | ,704^a^ | 0.495 | 0.476 | 574. 4 |
| 2 | ,807^b^ | 0.651 | 0.625 | 486.3 |
| a. Predictors: (Constant), Handgrip performance (FTI) at trial 1 | | | |  |
| b. Predictors: (Constant), Handgrip performance (FTI) at trial 1, Perceived temperature | | | |  |

Notes: FTI force time integral
